# Supplementary material for: Cell Therapy for Neurological Disorders: The Perspective of Promising Cells
Source: Biology (Basel). 2021 Nov 6;10(11):1142. doi: 10.3390/biology10111142 (PMC8614777; doi:10.3390/biology10111142)
Supplement: Supplementary file 1 [file biology-10-01142-s001.zip › biology-1430795-supplementary.pdf]

Table S1. Examples of hiPSC transplantation for neurological disorders in animal studies since 2016 (data from PubMed).

Subject	Disease model	Route of administration	Other supportive condition	Cell source	Follow up time (after transplantation)	Significant functional recovery	Reference
rat	Spinal cord injury	intraspinal or intravenous	None	SB5 hiPSC line	8 weeks	√	[1]
rat	hemorrhagic stroke	Intracerebral	None	Human fibroblast	4 weeks	√	[2]

Table S2: Eexamples of hNSPC transplantation for neurological disorders in animal studies since 2016 (data from PubMed).

Subject	Disease model	Route of administration	Other supportive condition	Cell source	Follow up time (after transplantation)	Significant functional recovery	Reference
mouse	SCI	Intraspinal	pre-treated with a gamma-secretase inhibitor	hiPSCs	4 months	√	[3]
mouse	SCI	Intraspinal	pre-treated with a gamma-secretase inhibitor	hiPSCs	89 days	√	[4]
rat	SCI	Intraspinal	region specific dI4 spinal NSPCs	hM3Dq-expressing hESCs (line WA09)	3 months	√	[5]
rat	SCI	Intraspinal	co-transplanted with MSCs and combined with a curcumin nanoconjugate	hiPSCs	8 weeks	×	[6]
rat	SCI	Intraspinal	seeded onto PuraMatrix peptide hydrogels	epileptic human brain specimens	6 weeks	√	[7]
mouse	SCI	Intraspinal	NSPCs with gliogenic competence	hiPSCs	12 weeks	√	[8]
mouse	SCI	Intraspinal	spinal cord-type NSPCs and forebrain-type NSPCs	hiPSCs	8 weeks	√ (in spinal cord-type NSPCs but not forebrain-type NSPCs	[9]
rat	SCI	Intraspinal	seeded onto aligned collagen sponge scaffolds	human fetal brain (hbNSPCs) and spinal cord- (hscNSPCs)	8 weeks	√ (in hscNSPC, but not hbNSPCs	[10]
rat	SCI	Intraspinal	none	urine-derived hiPSC	4 weeks	Not tested	[11]
rat	spinal post-traumatic cysts	Intraspinal	none	Human olfactory mucosa	4 weeks	√	[12]
rat	SCI	Intraspinal	GDNF-expressing NSPCs	hiPSCs	8 weeks	√	[13]
rat	SCI	Intraspinal	preconditioned with EI-tPA	hiPSCs	18 weeks	√	[14]
rat	SCI	Intraspinal	Co-transplantation with hUC-MSCs	Human fetal brain	2 weeks	√	[15]
rat	SCI	Intraspinal	combinational therapy with lithium	hESCs, cell line: Royan H6 line	7 weeks	√	[16]

mouse	SCI	Intraspinal	none	human fetal brain, cell line: hCNS-SCns	16 weeks	Not tested	[17]
rat	SCI	Intraspinal	loaded on longitudinal collagen sponge scaffolds	human fetal spinal cord	8 weeks	√ (better than hMSCs.)	[18]
mouse	HD	Intracerebral	none	Patient hiPSCs (HD72-iPSC)	12 weeks	√	[19]
rat	HD	Intracerebral	none	hiPSCs	11 weeks	√	[20]
rat	HD	Intracerebral	immortalized BDNF-overexpressing NSPCs	human fetal brain, cell line: HB1.F3.BDNF	7 weeks	√	[21]
rat	HD	Intracerebral	immortalized with the c-mycERTAM transgene	Human fetal cortex, cell line: CTX0E03	13 weeks	√	[22]
mouse	HD (R6/2 mice and Q140 mice)	Intracerebral	none	hESCs (Biotime ESI-017)	5 weeks for R6/2 mice and 6 months for Q140 mice	√	[23]
rat	MCAO-stroke	Intracerebral	none	HLA-homozygous hiPSCs	12 weeks	√	[24]
rat	MCAO-stroke	Intracerebral	none	urine-derived hiPSC line (UC-05)	35 days	√	[25]
mouse	Hypoxic-Ischemic Brain Injury	Intracerebral	pretreated with TNF-α	human fetal brain	9 weeks	√	[26]
rat	MCAO-stroke	Intracerebral	none	hiPSCs	12 weeks	√	[27]
mouse	MCAO-stroke	Intracranial	cell transplantation was combined with a delayed tPA administration	human fetuses	24 hours	Not tested	[28]
rat	MCAO-stroke	Intravenous	none	hiPSCs	2 months	√ (Better than MSCs)	[29]
rat	MCAO-stroke	Intracerebral	combined with repetitive transcranial magnetic stimulation	hESCs	31 days	√	[30]
rat	endothelin-1 focal ischemic stroke	Intracerebral	preconditioned with metformin	hiPSCs	7 weeks	√	[31]
rat	MCAO-stroke	Intra-epidural	none	hiPSCs	21 days	√	[32]
rat	Lacunar Stroke	Intracerebral	short-lived cells	human umbilical cord-blood	14 days	Not tested	[33]

rat	PD	Intracerebral	none	hESCs/hiPSCs derived midbrain organoids	9 months	√	[34]
rat	PD	Intracerebral	none	Human term placentas derived hMSCs	12 weeks	√	[35]
monkey	PD	Intracerebral	none	Human parthenogenetic stem cells (LLC2P)	12 months	√	[36]
mouse	AD	Intranasal	none	Human embryos	4 months	√	[37]
mouse	AD	Intracerebral or intravenous	none	Human fetal brain	3 months	√	[38]
mouse	AD	Intracranial	insulin-like growth factor-I (IGF-1)-expressing NSPCs	Human cortex, cell line: NSI-HK532-IGF-1	17 weeks	√	[39]
mouse	Dementia with Lewy Bodies	Intracerebral	none	Human fetal cortex, cell line: CNS10-hNPC	100 days	√	[40]
mouse	AD	Intracerebral	none	Human fetal brain	6 weeks	√	[41]
rat	Ataxia	Intracerebellar	none	Human fetal brain	70 days	√	[42]
rat	Ataxia	Intracerebellar	none	Human fetal brain	20 days	√	[43]
rat	Ataxia	Intracarotid or Intracerebral	none	Human fetal brain	18 days	√ (in intracerebral but not in intracarotid transplantation)	[44]
rat	TBI	Perilesional or intralesional	none	human fetal spinal cord, cell line: NSI-566	12 weeks	√ (perilesional transplantation was better)	[45]
rat	TBI	Intracerebral	none	Cell line: ISC-hpNSC	3 months	√	[46]
rat	TBI	Intracranial	seeded in PuraMatrix hydrogel	Epileptic human brain	28 days	√	[47]
mouse	TBI	Intracranial	none	hESCs derived NSPCs ((H1-DsRedNPCs)	5 weeks	√	[48]
rat	TBI with spontaneous seizures	Intracerebral	none	hESCs (Shef6)	14 weeks	√ (in rats with TBI but not in rats with both diseases)	[49]
rat	TBI	Intracerebral	none	hESCs (Shef6)	5 months	√	[50]
rat	ALS	Intracerebral	GDNF- expressing NSPCs	Human cortex, cell line: CNS10-hNPC-GDNF	4 months	√ (and extended survival)	[51]

Table S3. Examples of human neuron, oligodendrocyte, and astrocyte transplantation for neurological disorders in animal studies since 2016 (data from PubMed).

Cell type	Subject	Disease model	Route of administration	Other supportive condition	Cell source	Follow up time (after transplantation)	Significant functional recovery	Reference
Oligodendrocyte progenitors and motoneuron Progenitors	rat	SCI	Intraspinal	None	hESC (H9 and H1 lines)	4 months	√	[52]
Oligodendrocyte precursor cells	rat	SCI	Intraspinal	Seeded onto hydrogels which were modified with the RGD peptide and platelet-derived growth factor (PDGF-A)	hiPSCs	8 weeks	√	[53]
hiPSC-OPCs-enriched NSPCs	mouse	SCI	Intraspinal	None	hiPSCs (201B7)	12 weeks	√ (no significant difference with hiPSCs-NSPCs)	[54]
Motor neurons	mouse	ALS	Intrathecal	BDNF overexpressing-neurons	Human umbilical cord MSCs	1 month	√ (prolonged survival, did not delay disease onset)	[55]
Dopaminergic neurons	rat	PD	Intracerebral	None	Human chorionic MSCs	30 days	√	[56]
Dopaminergic neurons	monkey	PD	Intracerebral	None	Human Parthenogenetic ESCs (Q-CTS-hESC-1)	24 months	√	[57]
Dopaminergic neuron progenitors	monkey	PD	Intracerebral	None	hiPSCs	24 months	√	[58]
Dopaminergic neuron progenitors, immature dopaminergic neurons,	mouse	PD	Intracerebral	None	hESC line HES-3	3 months	√ (dopaminergic progenitors	[59]

or dopaminergic neurons							showed less capacity of producing functional recovery)	
Midbrain dopaminergic (mDA) neurons	mouse	PD	Intracerebral	DREADDs (designer receptors exclusively activated by designer drug)-expressing neurons	hESCs (line WA09	5 months	√	[60]
Dopaminergic-like neuron	rat	PD	Intracerebral	None	Human umbilical cord MSCs	6 weeks	√	[61]
Cholinergic-like neurons	rat	AD	Intracerebral	BDNF overexpressing-neurons	Human umbilical cord MSCs	8 weeks	√	[62]
Oligodendrocyte precursor cells (OPCs)	marmoset	MS	Intracortical	None	hiPSCs	40 days	Not tested	[63]

Table S4. Examples of hMSC transplantation for neurological disorders in animal studies since 2016 (data from PubMed).

Subject	Disease model	Route of administration	Other supportive condition	Cell source	Follow up time (after transplant ation)	Significant functional recovery	Reference
rat	SCI	Intraspinal	Combined with repetitive transcranial magnetic stimulation (rTMS)	Human umbilical cord blood	8 weeks	√	[64]
rat	SCI	Intraspinal	Enhanced gene expression of Wnt3a in hMSC	Human umbilical cord blood	8 weeks	√	[65]
canine	SCI	Intraspinal	Loaded on Collagen/heparin sulfate scaffolds	Human umbilical cord	6 months	√	[66]
mouse	SCI	Intraspinal	Loaded on dual-enzymatically cross-linked gelatin hydrogel with hydrogen horseradish peroxidase (HRP) and galactose oxidase (GalOx)	Human umbilical cord	42 days	√	[67]
rat	SCI	Intraspinal	Combined with an ultrashort wave therapy	Human umbilical cord	4 weeks	√ (ultrashort wave played a more important role)	[68]
rat	SCI	Intraspinal	Combined with a curcumin treatment	Human umbilical cord	9 weeks	√	[69]
rat and canine	Acute complete SCI	Intraspinal	Loaded on collagen scaffold	Human umbilical cord	6 months	√	[70]
rat	subacute incomplete SCI	Intrathecal	None	Human umbilical cord	30 days	√	[71]
rat	SCI	Intraspinal	Combined with photobiomodulation	Human umbilical cord	21 days	√	[72]
mouse	SCI	Intraspinal	None	Human umbilical cord	8 weeks	√	[73]

rat	SCI	Intracerebroventricular	None	Human umbilical cord	11 days	√	[74]
rat	contusive SCI	Intraspinal	Olig2-overexpressing hMSCs	Human bone marrow	7 weeks	√	[75]
mouse	SCI	Intraspinal	None	Human umbilical cord	8 weeks	√	[76]
rabbit	SCI	Intravenous	None	Human umbilical cord blood	8 weeks	√	[77]
rat	SCI	Intraspinal	Wnt3a-secreting hMSCs	Human umbilical cord blood	7 weeks	√	[78]
rat	SCI	Intraspinal	Combined with suppression of microRNA-383	Human bone marrow	28 days	√	[79]
rat	SCI	Intraspinal	None	Human amniotic membrane	28 days	√	[80]
rat	SCI	Intraspinal	Preconditioned in lowered oxygen	Human umbilical cord	4 weeks	√	[81]
rat	PD	Intracerebral	None	Human olfactory mucosa tissue	4 weeks	√	[82]
rat	PD	Intravenous	None	Human Wharton's jelly	21 weeks	√ (alone or in combination with L-Dopa)	[83]
mouse	PD	Intravenous	hMSCs were pre-activated by curcumin	Human umbilical cord	8 weeks	√	[84]
rat	PD	Intrathecal	Combined with a Repetitive transcranial magnetic stimulation treatment	Human bone marrow	4 weeks	√	[85]
mouse	PD	Intracranial	GDNF overexpressing-hMSCs	Human adipose	5 weeks	√	[85]
mouse	PD	Intranasal	None	Human endometrium	120 days	√	[86]
rat	PD	Intracerebral	Combined with physical exercise	Human adipose	54 days	√ (exercise played a more important role)	[87]
mouse	PD	Intravenous	Fibroblast growth factor-20-overexpressing hMSCs	Human umbilical cord	16 weeks	√	[88]

mouse	MCAO-stroke	Intraperitoneal	None	Human placenta	24 hours	√	[89]
rat	MCAO-stroke	Intravenous	C–C motif chemokine ligand 2 (CCL-2) overexpressing-hMSCs	Human umbilical cord	28 days	√	[90]
rat	MCAO-stroke	Intravenous	None	Human cranial bone or human iliac bone	28 days	√ (human cranial bone-derived MSCs were better)	[91]
rat	MCAO-stroke	Intracerebral	Neuregulin 1 overexpressing-hMSCs	Human adipose	13 days	√	[92]
rat	MCAO-stroke	Intra-arterial	Short-term preconditioning of hMSCs via 3D aggregation	Human bone marrow	4 weeks	√	[93]
rat	MCAO-stroke	Intracerebral	None	Human bone marrow	8 weeks	√	[94]
mouse	MCAO-stroke with Diabetes or hypertension	Intravenous	None	Human adipose	6 weeks	×	[95]
rat	MCAO-stroke	Intravenous	Combined with rehabilitation	Human adipose tissue	42 days	√	[96]
rat	MCAO-stroke	Intravenous	None	Human bone marrow, cell line: B10	13 days	Not tested	[97]
rat	MCAO-stroke	Intracerebral	None	Human turbinate or adipose tissue	13 days	√	[98]
rat	MCAO-stroke	Intravenous	None	Human umbilical cord	13 days	√	[99]
rat	MCAO-stroke	Intra-arterial	None	Human iliac crest	20 days	√ (young hMSCs were better)	[100]
rat	MCAO-stroke	Intravenous	None	Human Wharton's jelly	35 days	√	[101]
rat	acute Intracranial hemorrhage	Intracranial	Loaded on hyaluronic acid hydrogel scaffolds	hiPSCs	28 days	√	[102]
mouse	Intracerebral hemorrhage	Intravenous	None	Human adipose	Around 1 month	√	[103]

rat	Hemorrhagic Stroke	Intraventricular	None	Human bone marrow	21 days	√	[104]
rat	Intracerebral hemorrhage (ICH)	Intracerebral	Combined with Intracerebral injection of VII collagenase	Human amniotic membrane	27 days	√	[105]
mouse	TBI	Intranasal	Combined with a shCCL20-CCR6 nanodendriplexes treatment	Not mentioned	7 days	√	[106]
rat	TBI	Intravenous	None	Human umbilical cord	28 days	√	[107]
rat	TBI	Intravenous or Intranasal	Pioglitazone treatment prior to hMSCs transplantation	Not mentioned	30 days	√ (Intranasal administra tion showed higher number of hMSCs in the brain.)	[108]
rat	TBI	Intracerebral	CXCR4-overexpressing hMSCs were co-loaded with activated astrocytes on RADA16-BDNF peptide scaffold	Human umbilical cord	28 days	Not tested	[109]
mouse	ALS	Intramuscular	None	Human umbilical cord blood	11 weeks	√	[110]
mouse	Spinocerebellar ataxia type 1 (SCA1)	Intracerebellar	None	Human Wharton's jelly	5 months	√	[111]
monkey	MS	Intravenous	None	Human umbilical cord	66 days	√	[112]
mouse	AD	Intracerebral	None	Human menstrual blood	28 days	√	[113]
mouse	AD	Intravenous	Combined with resveratrol	Human umbilical cord	2 months	√	[114]
mouse	AD	Intravenous	None	Human umbilical cord	4 weeks	√	[115]
mouse	AD	Intracerebral	None	Human bone marrow	6 months	√	[116]
rat	epilepsy	Intracerebral	None	Human Wharton's jelly	28 days	√	[117]

Table S5. Examples of DPSC and SHED transplantation for neurological disorders in animal studies since 2016 (data from PubMed).

Cell type	Subject	Disease model	Route of administration	Other supportive condition	Follow up time (after transplantation)	Significant functional recovery	Reference
DPSCs	rat	SCI	Intraspinal	None	4 weeks	√	[118]
DPSCs	rat	SCI	Intraspinal	FGF2 pretreated-DPSCs	8 weeks	√ (in 3 of 4 cell lines)	[119]
DPSCs	rat	SCI	Intraspinal	FGF2 pretreated-DPSCs	7 weeks	√	[120]
DPSCs	rat	HD	Intracerebral	None	4 weeks	√	[121]
DPSCs	rat	cerebellar ataxia	Intracerebellar	None	30 days	√	[122]
DPSCs	rat	MCAO-stroke	Intravenous	None	72 hours	√	[123]
DPSCs	mouse	PD	Intrathecal	none	12 weeks	√	[124]
SHED	rat	SCI	Intraspinal	none	6 weeks	√	[125]
SHED	rat	SCI	Intraspinal	Combined with treadmill training	6 weeks	√ (treadmill training caused no functional improvement)	[126]
SHED	rat	stroke	Intracerebral or intravenous	None	1 month	√ (Intrahippocampal route showed better results.)	[127]

Table S6. Examples of human Muse cell transplantation for neurological disorders in animal studies since 2016 (data from PubMed).

Subject	Disease model	Route of administration	Other supportive condition	Cell source	Follow up time (after transplantation)	Significant functional recovery	Reference
mouse	lacunar stroke	Intracerebral	None	Human bone marrow	10 months	√	[128]
rat	MCAO-stroke	Intracerebral	None	Separated from normal human dermal fibroblasts	84 days	√	[129]
mouse	Intracerebral hemorrhage	Intracerebral	None	Separated from MSCs	69 days	√	[130]
rat	HIE	Intravenous	None	Separated from BM-MSCs	6 months	√	[131]
mouse	ALS	Intravenous	None	Separated from BM-MSCs	14 weeks	√	[132]
rat	SCI	Intravenous	None	CL2020, a human Muse cell-rich product	8 weeks	√	[133]

References

1. Bellak, T., et al. (2020). Grafted human induced pluripotent stem cells improve the outcome of spinal cord injury: modulation of the lesion microenvironment. *Sci Rep*. **10**(1): p. 22414.
2. Qin, J., et al. (2013). Functional recovery after transplantation of induced pluripotent stem cells in a rat hemorrhagic stroke model. *Neuroscience Letters*. **554**: p. 70-75.
3. Okubo, T., et al. (2018). Treatment with a Gamma-Secretase Inhibitor Promotes Functional Recovery in Human iPSC- Derived Transplants for Chronic Spinal Cord Injury. *Stem Cell Reports*. **11**(6): p. 1416-1432.
4. Okubo, T., et al. (2016). Pretreatment with a gamma-Secretase Inhibitor Prevents Tumor-like Overgrowth in Human iPSC-Derived Transplants for Spinal Cord Injury. *Stem Cell Reports*. **7**(4): p. 649-663.
5. Gong, C., et al. (2021). Human spinal GABA neurons alleviate spasticity and improve locomotion in rats with spinal cord injury. *Cell Rep*. **34**(12): p. 108889.

6. Bonilla, P., et al. (2021). Human-Induced Neural and Mesenchymal Stem Cell Therapy Combined with a Curcumin Nanoconjugate as a Spinal Cord Injury Treatment. *Int J Mol Sci*. **22**(11).
7. Abdolahi, S., et al. (2021). Improvement of Rat Spinal Cord Injury Following Lentiviral Vector-Transduced Neural Stem/Progenitor Cells Derived from Human Epileptic Brain Tissue Transplantation with a Self-assembling Peptide Scaffold. *Molecular Neurobiology*. **58**(6): p. 2481-2493.
8. Kamata, Y., et al. (2021). A robust culture system to generate neural progenitors with gliogenic competence from clinically relevant induced pluripotent stem cells for treatment of spinal cord injury. *Stem Cells Translational Medicine*. **10**(3): p. 398-413.
9. Kajikawa, K., et al. (2020). Cell therapy for spinal cord injury by using human iPSC-derived region-specific neural progenitor cells. *Molecular Brain*. **13**(1).
10. Zou, Y.L., et al. (2020). Aligned collagen scaffold combination with human spinal cord-derived neural stem cells to improve spinal cord injury repair. *Biomaterials Science*. **8**(18): p. 5145-5156.
11. Liu, A.M., et al. (2020). Transplantation of human urine-derived neural progenitor cells after spinal cord injury in rats. *Neuroscience Letters*. **735**.
12. Voronova, A.D., et al. (2020). Neural Stem/Progenitor Cells of Human Olfactory Mucosa for the Treatment of Chronic Spinal Cord Injuries. *Bull Exp Biol Med*. **168**(4): p. 538-541.
13. Khazaei, M., et al. (2020). GDNF rescues the fate of neural progenitor grafts by attenuating Notch signals in the injured spinal cord in rodents. *Sci Transl Med*. **12**(525).
14. Shiga, Y., et al. (2019). Tissue-type plasminogen activator-primed human iPSC-derived neural progenitor cells promote motor recovery after severe spinal cord injury. *Sci Rep*. **9**(1): p. 19291.
15. Sun, L., et al. (2019). Co-Transplantation of Human Umbilical Cord Mesenchymal Stem Cells and Human Neural Stem Cells Improves the Outcome in Rats with Spinal Cord Injury. *Cell Transplant*. **28**(7): p. 893-906.
16. Mohammadshirazi, A., et al. (2019). Combinational therapy of lithium and human neural stem cells in rat spinal cord contusion model. *Journal of Cellular Physiology*. **234**(11): p. 20742-20754.
17. Piltti, K.M., et al. (2017). Increasing Human Neural Stem Cell Transplantation Dose Alters Oligodendroglial and Neuronal Differentiation after Spinal Cord Injury. *Stem Cell Reports*. **8**(6): p. 1534-1548.
18. Zou, Y., et al. (2020). Comparison of Regenerative Effects of Transplanting Three-Dimensional Longitudinal Scaffold Loaded-Human Mesenchymal Stem Cells and Human Neural Stem Cells on Spinal Cord Completely Transected Rats. *ACS Biomater Sci Eng*. **6**(3): p. 1671-1680.
19. Jeon, I., et al. (2014). In Vivo Roles of a Patient-Derived Induced Pluripotent Stem Cell Line (HD72-iPSC) in the YAC128 Model of Huntington's Disease. *Int J Stem Cells*. **7**(1): p. 43-7.
20. Yoon, Y., et al. (2020). Neural Transplants From Human Induced Pluripotent Stem Cells Rescue the Pathology and Behavioral Defects in a Rodent Model of Huntington's Disease. *Frontiers in Neuroscience*. **14**.
21. Kim, H.S., et al. (2020). Intracerebral Transplantation of BDNF-overexpressing Human Neural Stem Cells (HB1.F3.BDNF) Promotes Migration, Differentiation and Functional Recovery in a Rodent Model of Huntington's Disease. *Exp Neurobiol*. **29**(2): p. 130-137.
22. Yoon, Y., et al. (2020). Implantation of the clinical-grade human neural stem cell line, CTX0E03, rescues the behavioral and pathological deficits in the quinolinic acid-lesioned rodent model of Huntington's disease. *Stem Cells*. **38**(8): p. 936-947.

23. Reidling, J.C., et al. (2018). Human Neural Stem Cell Transplantation Rescues Functional Deficits in R6/2 and Q140 Huntington's Disease Mice. *Stem Cell Reports*. **10**(1): p. 58-72.
24. Noh, J.E., et al. (2020). Intracerebral transplantation of HLA-homozygous human iPSC-derived neural precursors ameliorates the behavioural and pathological deficits in a rodent model of ischaemic stroke. *Cell Proliferation*. **53**(9).
25. Wu, R., et al. (2020). Transplantation of Neural Progenitor Cells Generated from Human Urine Epithelial Cell-Derived Induced Pluripotent Stem Cells Improves Neurological Functions in Rats with Stroke. *Discovery Medicine*. **29**(156): p. 53-64.
26. Kim, M., et al. (2020). TNF-alpha Pretreatment Improves the Survival and Function of Transplanted Human Neural Progenitor Cells Following Hypoxic-Ischemic Brain Injury. *Cells*. **9**(5).
27. Oh, S.H., et al. (2020). Multimodal Therapeutic Effects of Neural Precursor Cells Derived from Human-Induced Pluripotent Stem Cells through Episomal Plasmid-Based Reprogramming in a Rodent Model of Ischemic Stroke. *Stem Cells Int*. **2020**: p. 4061516.
28. Boese, A.C., et al. (2020). Human neural stem cells improve early stage stroke outcome in delayed tissue plasminogen activator-treated aged stroke brains. *Exp Neurol*. **329**: p. 113275.
29. Cherkashova, E.A., et al. (2019). Comparative Analysis of the Effects of Intravenous Administration of Placental Mesenchymal Stromal Cells and Neural Progenitor Cells Derived from Induced Pluripotent Cells on the Course of Acute Ischemic Stroke in Rats. *Bulletin of Experimental Biology and Medicine*. **166**(4): p. 558-566.
30. Peng, J.J., et al. (2019). Repetitive transcranial magnetic stimulation promotes functional recovery and differentiation of human neural stem cells in rats after ischemic stroke. *Experimental Neurology*. **313**: p. 1-9.
31. Ould-Brahim, F., et al. (2018). Metformin Preconditioning of Human Induced Pluripotent Stem Cell-Derived Neural Stem Cells Promotes Their Engraftment and Improves Post-stroke Regeneration and Recovery. *Stem Cells and Development*. **27**(16): p. 1085-1096.
32. Lee, I.H., et al. (2017). Delayed epidural transplantation of human induced pluripotent stem cell-derived neural progenitors enhances functional recovery after stroke. *Scientific Reports*. **7**.
33. Jablonska, A., et al. (2016). Short-Lived Human Umbilical Cord-Blood-Derived Neural Stem Cells Influence the Endogenous Secretome and Increase the Number of Endogenous Neural Progenitors in a Rat Model of Lacunar Stroke. *Molecular Neurobiology*. **53**(9): p. 6413-6425.
34. Kim, S.W., et al. (2021). Neural stem cells derived from human midbrain organoids as a stable source for treating Parkinson's disease: NSCs as a stable source for PD treatment. *Prog Neurobiol*: p. 102086.
35. Kim, H.W., et al. (2018). Dual Effects of Human Placenta-Derived Neural Cells on Neuroprotection and the Inhibition of Neuroinflammation in a Rodent Model of Parkinson's Disease. *Cell Transplantation*. **27**(5): p. 814-830.
36. Gonzalez, R., et al. (2016). Neural Stem Cells Derived From Human Parthenogenetic Stem Cells Engraft and Promote Recovery in a Nonhuman Primate Model of Parkinson's Disease. *Cell Transplantation*. **25**(11): p. 1945-1966.
37. Lu, M.H., et al. (2021). Intranasal Transplantation of Human Neural Stem Cells Ameliorates Alzheimer's Disease-Like Pathology in a Mouse Model. *Frontiers in Aging Neuroscience*. **13**.
38. Poltavtseva, R.A., et al. (2020). Effect of Transplantation of Neural Stem and Progenitor Cells on Memory in Animals with Alzheimer's Type Neurodegeneration. *Bull Exp Biol Med*. **168**(4): p. 589-596.

39. McGinley, L.M., et al. (2018). Human neural stem cell transplantation improves cognition in a murine model of Alzheimer's disease. *Scientific Reports*. **8**.
40. Goldberg, N.R.S., et al. (2017). Human Neural Progenitor Transplantation Rescues Behavior and Reduces alpha-Synuclein in a Transgenic Model of Dementia with Lewy Bodies. *Stem Cells Transl Med*. **6**(6): p. 1477-1490.
41. Li, X.Y., et al. (2016). Human Neural Stem Cell Transplantation Rescues Cognitive Defects in APP/PS1 Model of Alzheimer's Disease by Enhancing Neuronal Connectivity and Metabolic Activity. *Frontiers in Aging Neuroscience*. **8**.
42. Tierney, W.M., et al. (2020). Transplanted Human Neural Progenitor Cells Attenuate Motor Dysfunction and Lengthen Longevity in a Rat Model of Ataxia. *Cell Transplant*. **29**: p. 963689720920275.
43. Nuryyev, R.L., et al. (2017). Transplantation of Human Neural Progenitor Cells Reveals Structural and Functional Improvements in the Spastic Han-Wistar Rat Model of Ataxia. *Cell Transplantation*. **26**(11): p. 1811-1821.
44. Uhlendorf, T.L., et al. (2017). Efficacy of Two Delivery Routes for Transplanting Human Neural Progenitor Cells (NPCs) Into the Spastic Han-Wistar Rat, a Model of Ataxia. *Cell Transplantation*. **26**(2): p. 259-269.
45. Hu, Z., et al. (2020). Human neural stem cell transplant location-dependent neuroprotection and motor deficit amelioration in rats with penetrating traumatic brain injury. *J Trauma Acute Care Surg*. **88**(4): p. 477-485.
46. Lee, J.Y., et al. (2019). Human parthenogenetic neural stem cell grafts promote multiple regenerative processes in a traumatic brain injury model. *Theranostics*. **9**(4): p. 1029-1046.
47. Jahan-Abad, A.J., et al. (2018). Human Neural Stem/Progenitor Cells Derived From Epileptic Human Brain in a Self-Assembling Peptide Nanoscaffold Improve Traumatic Brain Injury in Rats. *Molecular Neurobiology*. **55**(12): p. 9122-9138.
48. Lin, G.Q., et al. (2018). Transplanted human neural precursor cells integrate into the host neural circuit and ameliorate neurological deficits in a mouse model of traumatic brain injury. *Neuroscience Letters*. **674**: p. 11-17.
49. Beretta, S., et al. (2017). Effects of Human ES-Derived Neural Stem Cell Transplantation and Kindling in a Rat Model of Traumatic Brain Injury. *Cell Transplantation*. **26**(7): p. 1247-1261.
50. Haus, D.L., et al. (2016). Transplantation of human neural stem cells restores cognition in an immunodeficient rodent model of traumatic brain injury. *Experimental Neurology*. **281**: p. 1-16.
51. Thomsen, G.M., et al. (2018). Transplantation of Neural Progenitor Cells Expressing Glial Cell Line-Derived Neurotrophic Factor into the Motor Cortex as a Strategy to Treat Amyotrophic Lateral Sclerosis. *Stem Cells*. **36**(7): p. 1122-1131.
52. Erceg, S., et al. (2010). Transplanted oligodendrocytes and motoneuron progenitors generated from human embryonic stem cells promote locomotor recovery after spinal cord transection. *Stem Cells*. **28**(9): p. 1541-9.
53. Fuhrmann, T., et al. (2016). Injectable hydrogel promotes early survival of induced pluripotent stem cell-derived oligodendrocytes and attenuates longterm teratoma formation in a spinal cord injury model. *Biomaterials*. **83**: p. 23-36.
54. Kawabata, S., et al. (2016). Grafted Human iPS Cell-Derived Oligodendrocyte Precursor Cells Contribute to Robust Remyelination of Demyelinated Axons after Spinal Cord Injury. *Stem Cell Reports*. **6**(1): p. 1-8.

55. Wang, J., et al. (2021). BDNF-overexpressing human umbilical cord mesenchymal stem cell-derived motor neurons improve motor function and prolong survival in amyotrophic lateral sclerosis mice. *Neurol Res*. **43**(3): p. 199-209.
56. Ebrahimi, V., et al. (2020). Functional dopaminergic neurons derived from human chorionic mesenchymal stem cells ameliorate striatal atrophy and improve behavioral deficits in Parkinsonian rat model. *Anat Rec (Hoboken)*. **303**(8): p. 2274-2289.
57. Wang, Y.K., et al. (2018). Human Clinical-Grade Parthenogenetic ESC-Derived Dopaminergic Neurons Recover Locomotive Defects of Nonhuman Primate Models of Parkinson's Disease. *Stem Cell Reports*. **11**(1): p. 171-182.
58. Kikuchi, T., et al. (2017). Human iPS cell-derived dopaminergic neurons function in a primate Parkinson's disease model. *Nature*. **548**(7669): p. 592-596.
59. Qiu, L., et al. (2017). Immature Midbrain Dopaminergic Neurons Derived from Floor-Plate Method Improve Cell Transplantation Therapy Efficacy for Parkinson's Disease. *Stem Cells Transl Med*. **6**(9): p. 1803-1814.
60. Chen, Y.J., et al. (2016). Chemical Control of Grafted Human PSC-Derived Neurons in a Mouse Model of Parkinson's Disease. *Cell Stem Cell*. **18**(6): p. 817-826.
61. Zhao, C., et al. (2016). Heat shock protein 60 affects behavioral improvement in a rat model of Parkinson's disease grafted with human umbilical cord mesenchymal stem cell-derived dopaminergic-like neurons. *Neurochemical Research*. **41**(6): p. 1238-1249.
62. Hu, W., et al. (2019). Brain-derived neurotrophic factor modified human umbilical cord mesenchymal stem cells-derived cholinergic-like neurons improve spatial learning and memory ability in Alzheimer's disease rats. *Brain Res*. **1710**: p. 61-73.
63. Thiruvalluvan, A., et al. (2016). Survival and Functionality of Human Induced Pluripotent Stem Cell-Derived Oligodendrocytes in a Nonhuman Primate Model for Multiple Sclerosis. *Stem Cells Transl Med*. **5**(11): p. 1550-1561.
64. Guo, M.G., et al. (2020). Enhancement of Neural Stem Cell Proliferation in Rats with Spinal Cord Injury by a Combination of Repetitive Transcranial Magnetic Stimulation (rTMS) and Human Umbilical Cord Blood Mesenchymal Stem Cells (hUCB-MSCs). *Medical Science Monitor*. **26**.
65. Yoon, H.H., et al. (2021). Optimal Ratio of Wnt3a Expression in Human Mesenchymal Stem Cells Promotes Axonal Regeneration in Spinal Cord Injured Rat Model. *J Korean Neurosurg Soc*.
66. Deng, W.S., et al. (2021). Collagen/heparin sulfate scaffold combined with mesenchymal stem cells treatment for canines with spinal cord injury: A pilot feasibility study. *J Orthop Surg (Hong Kong)*. **29**(2): p. 23094990211012293.
67. Yao, M., et al. (2021). Dual-enzymatically cross-linked gelatin hydrogel enhances neural differentiation of human umbilical cord mesenchymal stem cells and functional recovery in experimental murine spinal cord injury. *J Mater Chem B*. **9**(2): p. 440-452.
68. Na, L., et al. (2020). Ultrashort Wave Combined with Human Umbilical Cord Mesenchymal Stem Cell (HUC-MSC) Transplantation Inhibits NLRP3 Inflammasome and Improves Spinal Cord Injury via MK2/TTP Signalling Pathway. *Biomed Res Int*. **2020**: p. 3021750.
69. Wanjiang, W., et al. (2020). Curcumin Improves Human Umbilical Cord-Derived Mesenchymal Stem Cell Survival via ERK1/2 Signaling and Promotes Motor Outcomes After Spinal Cord Injury. *Cell Mol Neurobiol*.
70. Deng, W.S., et al. (2020). Collagen scaffold combined with human umbilical cord-mesenchymal stem cells transplantation for acute complete spinal cord injury. *Neural Regen Res*. **15**(9): p. 1686-1700.

71. Yang, Y., et al. (2020). Subarachnoid transplantation of human umbilical cord mesenchymal stem cell in rodent model with subacute incomplete spinal cord injury: Preclinical safety and efficacy study. *Exp Cell Res*. **395**(2): p. 112184.
72. Chen, H.L., et al. (2021). Effects of photobiomodulation combined with MSCs transplantation on the repair of spinal cord injury in rat. *Journal of Cellular Physiology*. **236**(2): p. 921-930.
73. Wu, L.L., et al. (2020). Repairing and Analgesic Effects of Umbilical Cord Mesenchymal Stem Cell Transplantation in Mice with Spinal Cord Injury. *Biomed Research International*. **2020**.
74. Nishida, F., et al. (2020). Intracerebroventricular Delivery of Human Umbilical Cord Mesenchymal Stem Cells as a Promising Therapy for Repairing the Spinal Cord Injury Induced by Kainic Acid. *Stem Cell Reviews and Reports*. **16**(1): p. 167-180.
75. Park, H.W., et al. (2018). Olig2 expressing Mesenchymal Stem Cells Enhance Functional Recovery after Contusive Spinal Cord Injury. *International Journal of Stem Cells*. **11**(2): p. 177-186.
76. Bao, C.S., et al. (2018). Transplantation of Human umbilical cord mesenchymal stem cells promotes functional recovery after spinal cord injury by blocking the expression of IL-7. *European Review for Medical and Pharmacological Sciences*. **22**(19): p. 6436-6447.
77. Yang, C.H., et al. (2018). Repeated injections of human umbilical cord blood-derived mesenchymal stem cells significantly promotes functional recovery in rabbits with spinal cord injury of two noncontinuous segments. *Stem Cell Research & Therapy*. **9**.
78. Seo, D.K., et al. (2017). Enhanced axonal regeneration by transplanted Wnt3a-secreting human mesenchymal stem cells in a rat model of spinal cord injury. *Acta Neurochirurgica*. **159**(5): p. 947-957.
79. Wei, G.J., et al. (2017). Suppression of MicroRNA-383 Enhances Therapeutic Potential of Human Bone-Marrow-Derived Mesenchymal Stem Cells in Treating Spinal Cord Injury via GDNF. *Cellular Physiology and Biochemistry*. **41**(4): p. 1435-1444.
80. Zhou, H.L., et al. (2016). Transplantation of Human Amniotic Mesenchymal Stem Cells Promotes Functional Recovery in a Rat Model of Traumatic Spinal Cord Injury. *Neurochemical Research*. **41**(10): p. 2708-2718.
81. Zhou, Z.L., et al. (2016). Preconditioning in lowered oxygen enhances the therapeutic potential of human umbilical mesenchymal stem cells in a rat model of spinal cord injury. *Brain Research*. **1642**: p. 426-435.
82. Farhadi, M., et al. (2021). Implantation of human olfactory ecto-mesenchymal stem cells restores locomotion in a rat model of Parkinson's disease. *J Chem Neuroanat*. **114**: p. 101961.
83. Jalali, M.S., et al. (2020). Transplanted Wharton's jelly mesenchymal stem cells improve memory and brain hippocampal electrophysiology in rat model of Parkinson's disease. *J Chem Neuroanat*. **110**: p. 101865.
84. Wang, Y.L., et al. (2020). Curcumin-Activated Mesenchymal Stem Cells Derived from Human Umbilical Cord and Their Effects on MPTP-Mouse Model of Parkinson's Disease: A New Biological Therapy for Parkinson's Disease. *Stem Cells International*. **2020**.
85. Lee, J.Y., et al. (2020). Combination of Human Mesenchymal Stem Cells and Repetitive Transcranial Magnetic Stimulation Enhances Neurological Recovery of 6-Hydroxydopamine Model of Parkinsonian's Disease. *Tissue Engineering and Regenerative Medicine*. **17**(1): p. 67-80.
86. Bagheri-Mohammadi, S., et al. (2019). Intranasal administration of endometrial mesenchymal stem cells as a suitable approach for Parkinson's disease therapy. *Molecular Biology Reports*. **46**(4): p. 4293-4302.

87. Cucarian, J.D., et al. (2019). Physical exercise and human adipose-derived mesenchymal stem cells ameliorate motor disturbances in a male rat model of Parkinson's disease. *Journal of Neuroscience Research*. **97**(9): p. 1095-1109.
88. Li, J.F., et al. (2016). The Effect of MSCs Derived from the Human Umbilical Cord Transduced by Fibroblast Growth Factor-20 on Parkinson's Disease. *Stem Cells International*. **2016**.
89. Barzegar, M., et al. (2021). Human placental mesenchymal stem cells improve stroke outcomes via extracellular vesicles-mediated preservation of cerebral blood flow. *EBioMedicine*. **63**: p. 103161.
90. Lee, S., et al. (2020). Enhancing the Therapeutic Potential of CCL2-Overexpressing Mesenchymal Stem Cells in Acute Stroke. *Int J Mol Sci*. **21**(20).
91. Oshita, J., et al. (2020). Early Transplantation of Human Cranial Bone-derived Mesenchymal Stem Cells Enhances Functional Recovery in Ischemic Stroke Model Rats. *Neurologia Medico-Chirurgica*. **60**(2): p. 83-93.
92. Ryu, S., et al. (2019). Therapeutic efficacy of neuregulin 1-expressing human adipose-derived mesenchymal stem cells for ischemic stroke. *PLoS One*. **14**(9): p. e0222587.
93. Yuan, X.G., et al. (2019). Aggregation of human mesenchymal stem cells enhances survival and efficacy in stroke treatment. *Cytotherapy*. **21**(10): p. 1033-1048.
94. Xie, P., et al. (2019). Therapeutic effect of transplantation of human bone marrow-derived mesenchymal stem cells on neuron regeneration in a rat model of middle cerebral artery occlusion. *Molecular Medicine Reports*. **20**(4): p. 3065-3074.
95. Mangin, G., et al. (2019). Intravenous Administration of Human Adipose Derived-Mesenchymal Stem Cells Is Not Efficient in Diabetic or Hypertensive Mice Subjected to Focal Cerebral Ischemia. *Front Neurosci*. **13**: p. 718.
96. Mu, J., et al. (2019). Combined Adipose Tissue-Derived Mesenchymal Stem Cell Therapy and Rehabilitation in Experimental Stroke. *Front Neurol*. **10**: p. 235.
97. Sheikh, A.M., et al. (2019). A Mesenchymal stem cell line (B10) increases angiogenesis in a rat MCAO model. *Experimental Neurology*. **311**: p. 182-193.
98. Lim, H., et al. (2018). Therapeutic Potential of Human Turbinate-Derived Mesenchymal Stem Cells in Experimental Acute Ischemic Stroke. *International Neurourology Journal*. **22**: p. S131-S138.
99. Lin, W., et al. (2017). Human Umbilical Cord Mesenchymal Stem Cells Preserve Adult Newborn Neurons and Reduce Neurological Injury after Cerebral Ischemia by Reducing the Number of Hypertrophic Microglia/Macrophages. *Cell Transplantation*. **26**(11): p. 1798-1810.
100. Yamaguchi, S., et al. (2018). Age of donor of human mesenchymal stem cells affects structural and functional recovery after cell therapy following ischaemic stroke. *Journal of Cerebral Blood Flow and Metabolism*. **38**(7): p. 1199-1212.
101. Zhang, L., et al. (2017). Neural differentiation of human Wharton's jelly-derived mesenchymal stem cells improves the recovery of neurological function after transplantation in ischemic stroke rats. *Neural Regeneration Research*. **12**(7): p. 1103-1110.
102. Chen, K.H., et al. (2019). Human induced pluripotent stem cell-derived mesenchymal stem cell therapy effectively reduced brain infarct volume and preserved neurological function in rat after acute intracranial hemorrhage. *American Journal of Translational Research*. **11**(9): p. 6232-6248.
103. Kuramoto, Y., et al. (2019). Intravenous administration of human adipose-derived stem cells ameliorates motor and cognitive function for intracerebral hemorrhage mouse model. *Brain Res*. **1711**: p. 58-67.

104. Huang, P., et al. (2019). Safety and Efficacy of Intraventricular Delivery of Bone Marrow-Derived Mesenchymal Stem Cells in Hemorrhagic Stroke Model. *Sci Rep*. **9**(1): p. 5674.
105. Zhou, H.L., et al. (2016). Transplantation of human amniotic mesenchymal stem cells promotes neurological recovery in an intracerebral hemorrhage rat model. *Biochemical and Biophysical Research Communications*. **475**(2): p. 202-208.
106. Mayilsamy, K., et al. (2020). Treatment with shCCL20-CCR6 nanodendriplexes and human mesenchymal stem cell therapy improves pathology in mice with repeated traumatic brain injury. *Nanomedicine-Nanotechnology Biology and Medicine*. **29**.
107. Chen, K.H., et al. (2020). Human Umbilical Cord-Derived Mesenchymal Stem Cell Therapy Effectively Protected the Brain Architecture and Neurological Function in Rat After Acute Traumatic Brain Injury. *Cell Transplantation*. **29**.
108. Das, M., et al. (2019). Pioglitazone treatment prior to transplantation improves the efficacy of human mesenchymal stem cells after traumatic brain injury in rats. *Scientific Reports*. **9**.
109. Huda, F., et al. (2016). Fusion of Human Fetal Mesenchymal Stem Cells with "Degenerating" Cerebellar Neurons in Spinocerebellar Ataxia Type 1 Model Mice. *Plos One*. **11**(11).
110. Kook, M.G., et al. (2020). Repeated intramuscular transplantations of hUCB-MSCs improves motor function and survival in the SOD1 G(93)A mice through activation of AMPK. *Scientific Reports*. **10**(1).
111. Tsai, P.J., et al. (2019). Xenografting of human umbilical mesenchymal stem cells from Wharton's jelly ameliorates mouse spinocerebellar ataxia type 1. *Translational Neurodegeneration*. **8**(1).
112. Liu, S., et al. (2019). Therapeutic effect of transplanted umbilical cord mesenchymal stem cells in a cynomolgus monkey model of multiple sclerosis. *Am J Transl Res*. **11**(4): p. 2516-2531.
113. Zhao, Y.J., et al. (2018). Transplantation of Human Menstrual Blood-Derived Mesenchymal Stem Cells Alleviates Alzheimer's Disease-Like Pathology in APP/PS1 Transgenic Mice. *Frontiers in Molecular Neuroscience*. **11**.
114. Wang, X.X., et al. (2018). Resveratrol promotes hUC-MSCs engraftment and neural repair in a mouse model of Alzheimer's disease. *Behavioural Brain Research*. **339**: p. 297-304.
115. Cui, Y.B., et al. (2017). Human umbilical cord mesenchymal stem cells transplantation improves cognitive function in Alzheimer's disease mice by decreasing oxidative stress and promoting hippocampal neurogenesis. *Behavioural Brain Research*. **320**: p. 291-301.
116. Ruzicka, J., et al. (2016). Mesenchymal Stem Cells Preserve Working Memory in the 3xTg-AD Mouse Model of Alzheimer's Disease. *International Journal of Molecular Sciences*. **17**(2).
117. Huang, P.Y., et al. (2016). Xenograft of human umbilical mesenchymal stem cells from Wharton's jelly as a potential therapy for rat pilocarpine-induced epilepsy. *Brain Behavior and Immunity*. **54**: p. 45-58.
118. Kabatas, S., et al. (2018). Neuronal regeneration in injured rat spinal cord after human dental pulp derived neural crest stem cell transplantation. *Bratislava Medical Journal-Bratislavske Lekarske Listy*. **119**(3): p. 143-151.
119. Sugiyama, K., et al. (2019). FGF2-responsive genes in human dental pulp cells assessed using a rat spinal cord injury model. *J Bone Miner Metab*. **37**(3): p. 467-474.

120. Nagashima, K., et al. (2017). Priming with FGF2 stimulates human dental pulp cells to promote axonal regeneration and locomotor function recovery after spinal cord injury. *Sci Rep*. **7**(1): p. 13500.
121. Eskandari, N., et al. (2020). Transplantation of human dental pulp stem cells compensates for striatal atrophy and modulates neuro-inflammation in 3-nitropropionic acid rat model of Huntington's disease. *Neurosci Res*.
122. Aliaghaei, A., et al. (2019). Dental pulp stem cell transplantation ameliorates motor function and prevents cerebellar atrophy in rat model of cerebellar ataxia. *Cell Tissue Res*. **376**(2): p. 179-187.
123. Nito, C., et al. (2018). Transplantation of human dental pulp stem cells ameliorates brain damage following acute cerebral ischemia. *Biomed Pharmacother*. **108**: p. 1005-1014.
124. Gnanasegaran, N., et al. (2017). Effect of dental pulp stem cells in MPTP-induced old-aged mice model. *Eur J Clin Invest*. **47**(6): p. 403-414.
125. Nicola, F., et al. (2019). Stem Cells from Human Exfoliated Deciduous Teeth Modulate Early Astrocyte Response after Spinal Cord Contusion. *Mol Neurobiol*. **56**(1): p. 748-760.
126. Nicola, F.C., et al. (2016). Human dental pulp stem cells transplantation combined with treadmill training in rats after traumatic spinal cord injury. *Braz J Med Biol Res*. **49**(9): p. e5319.
127. Zhu, S., et al. (2020). Transplantation of Stem Cells from Human Exfoliated Deciduous Teeth Decreases Cognitive Impairment from Chronic Cerebral Ischemia by Reducing Neuronal Apoptosis in Rats. *Stem Cells Int*. **2020**: p. 6393075.
128. Uchida, H., et al. (2017). Human Muse Cells Reconstruct Neuronal Circuitry in Subacute Lacunar Stroke Model. *Stroke*. **48**(2): p. 428-435.
129. Uchida, H., et al. (2016). Transplantation of Unique Subpopulation of Fibroblasts, Muse Cells, Ameliorates Experimental Stroke Possibly via Robust Neuronal Differentiation. *Stem Cells*. **34**(1): p. 160-173.
130. Shimamura, N., et al. (2017). Neuro-regeneration therapy using human Muse cells is highly effective in a mouse intracerebral hemorrhage model. *Experimental Brain Research*. **235**(2): p. 565-572.
131. Suzuki, T., et al. (2020). Intravenously delivered multilineage-differentiating stress enduring cells dampen excessive glutamate metabolism and microglial activation in experimental perinatal hypoxic ischemic encephalopathy. *J Cereb Blood Flow Metab*: p. 271678X20972656.
132. Yamashita, T., et al. (2020). Therapeutic benefit of Muse cells in a mouse model of amyotrophic lateral sclerosis. *Sci Rep*. **10**(1): p. 17102.
133. Kajitani, T., et al. (2021). Association of intravenous administration of human Muse cells with deficit amelioration in a rat model of spinal cord injury. *J Neurosurg Spine*: p. 1-8.
